# Four Common Vascular Endothelial Growth Factor Polymorphisms (−2578C>A, −460C>T, +936C>T, and +405G>C) in Susceptibility to Lung Cancer: A Meta-Analysis

**DOI:** 10.1371/journal.pone.0075123

**Published:** 2013-10-01

**Authors:** Ling Lin, Kejian Cao, Wenhu Chen, Xufeng Pan, Heng Zhao

**Affiliations:** Department of Thoracic Surgery, Shanghai Chest Hospital, School of Medicine, Shanghai Jiaotong University, Shanghai, China; University of Colorado, Denver, United States of America

## Abstract

**Background and Objective:**

Vascular endothelial growth factor (VEGF) is one of the key initiators and regulators of angiogenesis and it plays a vital role in the onset and development of malignancy. The association between *VEGF* gene polymorphisms and lung cancer risk has been extensively studied in recent years, but currently available results remain controversial or ambiguous. The aim of this meta-analysis is to investigate the associations between four common *VEGF* polymorphisms (i.e., −2578C>A, −460C>T, +936C>T and +405C>G) and lung cancer risk.

**Methods:**

A comprehensive search was conducted to identify all eligible studies to estimate the association between *VEGF* polymorphisms and lung cancer risk. Crude odds ratios (ORs) with 95% confidence intervals (CIs) were used to evaluate the strength of this association.

**Results:**

A total of 14 published case-control studies with 4,664 cases and 4,571 control subjects were identified. Our meta-analysis provides strong evidence that *VEGF* −2578C>A polymorphism is capable of increasing lung cancer susceptibility, especially among smokers and lung squamous cell carcinoma (SCC) patients. Additionally, for +936C>T polymorphism, increased lung cancer susceptibility was only observed among lung adenocarcinoma patients. In contrast, *VEGF* −460C>T polymorphism may be a protective factor among nonsmokers and SCC patients. Nevertheless, we did not find any association between +405C>G polymorphism and lung cancer risk, even when the groups were stratified by ethnicity, smoking status or histological type.

**Conclusion:**

This meta-analysis recommends more investigations into the relationship between −2578C>A and −460C>T lung cancer risks. More detailed and well-designed studies should be conducted to identify the causal variants and the underlying mechanisms of the possible associations.

## Introduction

Lung cancer, characterized by uncontrolled cell growth in tissues of the lung [1], accounts for 13% (1.6 million) of the total cancer cases and 18% (1.4 million) of total deaths in 2008 [2]. Lung cancer has become a major public health challenge all over the world, especially in China [3]. Thus, understanding the molecular biology and etiology of lung cancer will be pivotal in designing targeted therapies and personalized medicines. The link to smoking as a definite causative agent for lung cancer has been well established from epidemiologic evidence since the 1950s [4], [5]. However, epidemiological data showed that only 10–15% of heavy tobacco smokers ultimately develop lung cancer [6], [7], suggesting that certain common genetic variants or polymorphisms may influence the risk of lung cancer, particularly among those who have developed lung cancer. Vascular endothelial growth factor (VEGF), also known as vascular permeability factor, is one of the key initiators and regulators of angiogenesis and it plays a critical role in the progress and prognosis of malignancy [8]–[10]. Evidence from *in vitro* and *in vivo* experiments have shown that high levels of VEGF expression were found to be associated with tumor growth and metastasis, whereas the inhibition of VEGF signaling results in suppression of both tumor-induced angiogenesis and tumor growth [11]–[13]. Bevacizumab, one of the agents for recognizing and blocking vascular endothelial growth factor A (VEGF-A), has been a promising agent in a combination regimen in improving the overall survival and progression-free survival of breast cancer, non-small-cell lung cancer, renal cell carcinoma, and other solid malignancies [14], [15].

The *VEGF* gene, which contains a 14-kb coding region with eight exons and seven introns, is located on chromosome 6p21.3 [16]. At least 30 single nucleotide polymorphisms (SNPs) in *VEGF* gene have been identified and described, and some have even been shown to affect the expression of *VEGF* gene [17], [18]. Several previously published meta-analyses showed that *VEGF* +936C>T (rs3025039), one of the most common polymorphisms, was not associated with gastric cancer [19]–[21], colorectal cancer [22], or breast cancer [23]–[25]. Additionally, these published meta-analyses also showed that other three common *VEGF* polymorphisms, −1154G>A (rs1570360), −634G>C (rs2010963) and −460C>T (rs833061), were not associated with colorectal cancer [26] or breast cancer [24], whereas the *VEGF* −634G/C polymorphism was found to be associated with gastric cancer [20]. In recent years, four common polymorphisms in *VEGF* gene, −2578C>A, −460C>T, +936C>T, and +405C>G, have been described in several literatures to appear to be involved in the development of lung cancer [27]–[31]. However, the results remain controversial or inconclusive. To the best of our knowledge, there were no published meta-analyses investigating the association between *VEGF* gene polymorphisms and lung cancer susceptibility. Therefore, we performed a meta-analysis of all eligible case-control or cohort studies to investigate whether these functional *VEGF* polymorphisms are associated with any increased risk of lung cancer and whether the associations are modulated by smoking status, histological type or other risk factors. We hope our meta-analysis can potentially be important in early lung cancer identification and become part of the therapeutic strategies in combating lung cancer.

## Materials and Methods

### Literature Search

Relevant papers for this meta-analysis were systematically identified through literature searches on PubMed, Embase, Web of science and Chinese National Knowledge Infrastructure (CNKI), and Chinese Biomedical Literature Database (CBM) of publications published up to March 9, 2013 relating to *VEGF* gene polymorphisms and lung cancer risk. As the main search criteria, we used combinations of the following terms: "*VEGF*", "vascular endothelial growth factor A", "vascular permeability factor", "vascular endothelial growth factor", "lung neoplasms", "pulmonary neoplasms", "bronchial neoplasms", "lung cancer", "bronchial neoplasm", "genetic polymorphism", "single nucleotide polymorphism", "SNP", "mutant", "gene variation". We also reviewed the reference lists of articles retrieved to identify relevant publications.

### Inclusion and Exclusion Criteria

Our meta-analysis included genetic association studies fulfilling the following inclusion criteria: (a) a case-control, cohort or cross-sectional study must evaluated at least one of four polymorphisms of *VEGF* gene and lung cancer risk; (b) the diagnosis of lung cancer patients was confirmed pathologically and controls were confirmed as cancer-free patients; (c) inclusion of sufficient data on the size of the sample, odds ratio (OR), and 95% confidence interval (CI) and (d) articles were published in the English or Chinese language.

Studies were excluded when they represented duplicates of previous publications, or were meta-analyses, case report, letters, reviews or editorial articles. Studies investigating the progression, severity, phenotype modification, response to treatment, or survival were also excluded. Additionally, when data was included in multiple studies using the same case series, either the study with the largest sample size or most recent publication was selected. Finally, family-based studies were excluded because of different design settings. Any disagreements on study inclusion were resolved through discussions between the authors. To ensure the rigour of the current meta-analysis, it was designed and reported according to the Preferred Reporting Items for Systematic Reviews and Meta-analyses (PRISMA) statement. The relevant checklist is shown in Supplement S1.

### Data Extraction

All data from the included studies were extracted independently by two investigators, using a piloted data standardized form (when it came to conflicting evaluations, an agreement was settled after a discussion): the first author’s surname, year of publication, country of origin, published language, gender of study individuals and ethnic subgroups, study design, number of subjects, smoking status, histological types of lung cancer, SNP genotyping methods, genotyping method and detected sample, allele and genotype frequencies, and evidence of Hardy-Weinberg equilibrium (HWE) in controls. In addition, we also compared key study characteristics such as location, study time and authorship to determine the existence of multiple publications from the same study.

### Quality Assessment of Included Studies

Two authors independently assessed the quality of the published articles according to the modified STROBE quality score systems [32]. Forty assessment items matching with the quality appraisals were used in this meta-analysis, with scores ranging from 0 to 40. Scores of 0–20, 20–30 and 30–40 were defined as low, moderate and high quality, respectively. The two authors resolved their differences through discussions; if no agreement could be reached, a third author decided on a decision. The modified STROBE quality score system is available in Supplement S2.

### Statistical Analysis

Crude ORs together with their corresponding 95% CIs were used to calculate and assess the strength of association between *VEGF* gene polymorphisms and lung cancer risk under five genetic models: allele, dominant, recessive, homozygous, and heterozygous models. The deviation of frequency from those expected under Hardy-Weinberg equilibrium (HWE) was assessed by Chi-squared goodness of fit tests in controls. We explored inter-study variation through prespecified subgrouping of studies according to ethnicity (ie, Caucasian or Asian), gender (ie, female or male), smoking status (ie, smoker or non-smoker), and histological type of lung cancer (ie, adenocarcinoma, squamous cell carcinoma (SCC), and small cell carcinoma (SCLC), where applicable. The statistical significance of the pooled OR was assessed with a Z test. Between-study variation and heterogeneity were estimated using Cochran’s *Q*-statistic, with *P*<0.05 as a cutoff for statistically significant heterogeneity [33].

We also quantified the effect of heterogeneity with the *I^2^* test (ranges from 0 to 100%), which represents the proportion of inter-study variability that can be attributed to heterogeneity rather than to chance [34]. The fixed effects model (*Mantel-Haenszel* method) was used, except when a significant *Q*-test (*P*<0.05) or *I^2^*>50% indicated the existence of heterogeneity among studies; otherwise, the random effects model (*DerSimonian-Laird* method) was applied for meta-analysis. In order to ensure the reliability of our results, sensitivity analysis was performed by omitting individual studies. Begger’s funnel plots were used to detect publication bias. In addition, Egger’s linear regression test, which measures funnel plot asymmetry via a natural logarithm scale of OR, was also used to evaluate publication bias [35]. All P-values were two-sided. Analyses were conducted with STATA Version 12.0 software (Stata Corp, College Station, TX).

## Results

### The Characteristics of Included Studies

**Figure 1:**
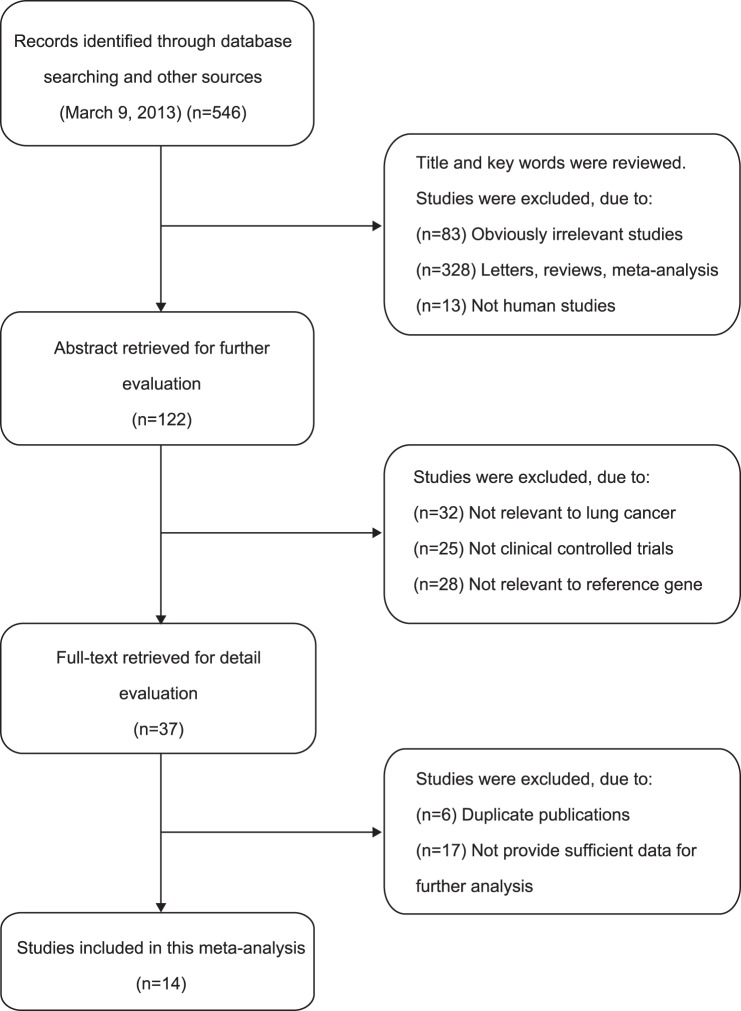
Flow diagram of the selection process of the studies and specific reasons for exclusion from the present meta-analysis.

Our initial literature search yielded 546 reports, which included 13 population-based [28]–[31], [36]–[44] and one hospital-based [27] case-control studies meeting the inclusion criteria based on the search criteria for lung cancer susceptibility linking to at least one of four common SNPs of *VEGF* gene, −2578C>A, −460C>T, +936C>T, and +405C>G. The flow diagram of the selection of studies and specific reasons for exclusion from the meta-analysis are shown in Figure 1. We studied four *VEGF* SNPs in 4,664 unrelated lung cancer cases and 4,571 unrelated controls from 14 case-control studies. In the eligible studies, there were 12 studies of subjects of Asian descent and only two studies of subjects of Caucasian descent. All included studies extracted DNA from peripheral blood and the *VEGF* polymorphisms were determined by classic PCR-RFLP in 12 studies, by TaqMan in 1 study, and by PIRA-PCR in another study. SNP genotypes were tested for departures from HWE for controls and all SNPs were in HWE. The qualities of the included studies were moderately high, with a STROBE score of greater than 20. The selected study characteristics were summarized in Table 1. The evaluation of the associations between *VEGF* −2578C>A, −460C>T, +936C>T, and +405C>G polymorphisms and lung cancer risk are presented in Tables 2, 3, 4 and 5.

**Table 1:** Main characteristics and methodological quality of all eligible studies.

Firstauthor	Year	Country	Ethnicity	Simple size(Case/Control)	Enrolledpatients	Sourceofcontrol	Characteristicsof controls(matched for)	Sample	Genotypemethod	SNP ID	Aliasname	STROBEscores
Lee et al	2005	Korea	Caucasian	432/432	Lungcancer	PCC	Patients(Age and gender)	Blood	PCR-RFLP	rs3025039	+936 C/T	27/40
										rs833061	−460 C/T	
										rs2010963	+405 C/G	
Zhai et al	2008	USA	Caucasian	1900/1458	NSCLC	HCC	Patients (residenceand ethnicity)	Blood	Taqman	rs3025039	+936 C/T	25/40
										rs833061	−460 C/T	
										rs2010963	+405 C/G	
Tan et al	2008	China	Asian	276/279	NSCLC	PCC	Healthy	Blood	PCR-RFLP	rs3025039	+936 C/T	21/40
										rs699947	−2578 C/A	
Wang et al	2008	China	Asian	377/416	Lungcancer	PCC	Healthy	Blood	PCR-RFLP	rs699947	−2578 C/A	20/40
Liang et al	2009	China	Asian	171/172	Lungcancer	PCC	Healthy (age,gender, andethnicity)	Blood	PCR-RFLP	rs3025039	+936 C/T	23/40
										rs699947	−2578 C/A	
Liu et al	2010	China	Asian	172/184	Lungcancer	PCC	Healthy (residenceand ethnicity)	Blood	PCR-RFLP	rs699947	−2578 C/A	22/40
										rs2010963	+405 C/G	
Yuan et al	2011	China	Asian	251/255	Lungcancer	PCC	Healthy (residenceand ethnicity)	Blood	PIRA-PCR	rs699947	−2578 C/A	21/40
									PCR-RFLP	rs833061	−460 C/T	
Li et al	2012a	China	Asian	150/150	Lungcancer	PCC	Healthy (age,gender, andethnicity)	Blood	PCR-RFLP	rs3025039	+936 C/T	25/40
										rs699947	−2578 C/A	
Naik et al	2012	India	Asian	100/150	Lungcancer	PCC	Healthy (age,gender, andethnicity)	Blood	PCR-RFLP	rs3025039	+936 C/T	26/40
										rs2010963	+405 C/G	
Gao et al	2012	China	Asian	200/204	Lungcancer	PCC	Healthy (age,residence andethnicity)	Blood	PCR-RFLP	rs833061	−460 C/T	23/40
Li et al	2012b	China	Asian	50/50	Lungcancer	PCC	Healthy (age,residence andethnicity)	Blood	PCR-RFLP	rs3025039	+936 C/T	21/40
Liu et al	2012	China	Asian	260/260	Lungcancer	PCC	Healthy (age,residence andethnicity)	Blood	PCR-RFLP	rs833061	−460 C/T	23/40
										rs2010963	+405 C/G	
Naykoo et al	2013	India	Asian	199/401	Lungcancer	PCC	Healthy (age,residence andethnicity)	Blood	RT-PCR	rs3025039	+936 C/T	27/40
										rs699947	−2578 C/A	
										rs2010963	+405 C/G	
Sun et al	2013	China	Asian	126/160	Lungcancer	PCC	Healthy (age,gender, andethnicity)	Blood	PCR-RFLP	rs833061	−460 C/T	26/40

Abbreviations: NSCLC, non-small cell lung cancer; PCC, population-based case-control study; HCC, hospital-based case–control study; PCR, polymerase chain reaction; RFLP, restriction fragment length polymorphism; PIRA, primer-introduced restriction analysis; SNPs, single nucleotide polymorphisms.

**Table 2:** Subgroup analyses for the associations of VEGF −2578C/A with susceptibility to lung cancer in Asians.

Subgroups	No. ofcase/control	Allele model(A allele vs. C allele)		Dominantmodel(AA+AC vs. CC)		Recessivemodel(AA vs. CC+AC)		Homozygousmodel(AA vs. CC)		Heterozygousmodel(AA vs. AC)	
		OR(95%CI)	*P*value	OR(95%CI)	*P*value	OR(95%CI)	*P*value	OR(95%CI)	*P*value	OR(95%CI)	*P*value
*Smoking status*											
Smoker	583/221	2.18(1.55–3.09)	<0.001	1.67(1.17–2.39)	0.005	5.78(1.70–19.6)	0.005	7.31(2.14–25.0)	0.002	3.71(1.06–13.0)	0.041
Non-smoker	434/390	1.20(0.89–1.63)	0.236	1.11(0.81–1.53)	0.525	1.02(0.43–2.45)	0.962	1.19(0.48–2.92)	0.703	0.85(0.34–2.10)	0.726
*Histological type*											
Adenocarcinoma	305/611	1.46(1.04–2.04)	0.028	1.23(0.88–1.72)	0.221	1.08(0.35–3.33)	0.892	1.40(0.44–4.40)	0.568	0.78(0.25–2.46)	0.674
SCC	374/611	1.90(1.43–2.52)	<0.001	1.39(1.01–1.90)	0.041	4.87(2.47–9.61)	<0.001	5.64(2.79–11.4)	<0.001	3.97(1.94–8.10)	<0.001
SCLC	152/611	1.10(0.72–1.70)	0.654	0.96(0.60–1.54)	0.859	0.88(0.20–3.92)	0.863	0.93(0.21–4247)	0.928	0.80(0.17–3.69)	0.771
*Country of origin*											
China	1397/1456	1.25(1.05–1.49)	0.014	1.15(0.84–1.59)	0.378	1.88(1.27–2.79)	0.002	2.00(1.42–2.81)	<0.001	2.06(1.07–3.97)	0.032
India	199/401	1.75(1.37–2.24)	<0.001	1.26(4.54–11.6)	<0.001	1.12(0.26–5.10)	0.163	1.07(0.24–4.87)	0.128	1.08(1.03–1.19)	<0.001
Overall	1596/1857	1.31(1.10–1.57)	0.003	1.46(0.88–2.42)	0.141	1.47(0.71–3.08)	0.298	1.79(1.30–2.46)	<0.001	1.37(0.50–3.76)	0.534

Abbreviations: OR, odds ratio; CI: confidence intervals.

**Table 3:** Subgroup analyses for the associations of VEGF −460C/T with susceptibility to lung cancer.

Subgroups	No. ofcase/control	Allele model(T allele vs.C allele)		Dominantmodel(TT+CT vs. CC)		Recessivemodel(TT vs. CC+CT)		Homozygousmodel(TT vs. CC)		Heterozygousmodel(TT vs. CT)	
		OR(95%CI)	*P*value	OR(95%CI)	*P*value	OR(95%CI)	*P*value	OR(95%CI)	*P*value	OR(95%CI)	*P*value
*Ethnicity*											
Caucasian(USA)	2332/1890	0.99(0.91–1.09)	0.951	1.12(0.80–1.55)	0.516	0.96(0.84–1.10)	0.559	1.06(0.81–1.39)	0.679	0.94(0.82–1.08)	0.382
Asian(China)	837/879	0.90(0.64–1.28)	0.572	0.67(0.38–1.19)	0.170	1.00(0.63–1.59)	0.990	0.72(0.35–1.49)	0.373	1.09(0.68–1.75)	0.729
*Smoking status*											
Smoker	842/303	0.86(0.68–1.09)	0.224	0.85(0.52–1.39)	0.519	0.84(0.62–1.13)	0.247	0.83(0.49–1.40)	0.474	0.85(0.61–1.17)	0.322
Non-smoker	580/416	0.65(0.42–1.01)	0.056	0.32(0.18–0.55)	<0.001	0.73(0.34–1.58)	0.423	0.35(0.20–0.61)	<0.001	0.88(0.37–2.07)	0.767
*Histological type*											
Adenocarcinoma	676/1151	0.86(0.72–1.04)	0.116	0.62(0.41–0.93)	0.021	0.91(0.72–1.16)	0.458	0.70(0.45–1.09)	0.116	0.98(0.76–1.26)	0.863
SCC	843/1151	0.81(0.68–0.96)	0.013	0.71(0.48–1.04)	0.077	0.78(0.63–0.97)	0.026	0.69(0.46–0.94)	0.015	0.81(0.65–0.99)	0.020
SCLC	356/1151	0.96(0.75–1.23)	0.759	0.81(0.46–1.42)	0.454	1.00(0.74–1.36)	0.999	0.89(0.50–1.60)	0.699	1.04(0.75–1.44)	0.807
Overall	3167/2769	0.94(0.79–1.12)	0.495	0.86(0.59–1.24)	0.415	0.98(0.73–1.32)	0.788	0.89(0.60–1.33)	0.579	1.01(0.79–1.28)	0.969

Abbreviations: OR, odds ratio; CI, confidence intervals.

**Table 4:** Subgroup analyses for the associations of VEGF +936C/T with susceptibility to lung cancer under dominant model.

Subgroups	No. ofcase/control	Dominant model(TT+CT vs. CC)	
		OR (95%CI)	*P* value
*Ethnicity*			
Caucasian	2332/1890	0.99 (0.80–1.22)	0.937
Asian	856/1202	1.28 (0.77–2.12)	0.338
*Gender*			
Male	1203/784	1.00 (0.81–1.23)	0.972
Female	1018/966	1.12 (0.92–1.38)	0.266
*Smoking status*			
Smoker	155/64	0.74 (0.39–1.41)	0.360
Non-smoker	166/258	1.56 (1.01–2.43)	0.052
*Histological type*			
Adenocarcinoma	310/754	1.24 (1.03–1.44)	0.012
SCC	316/754	0.98 (0.73–1.29)	0.858
SCLC	113/754	0.45 (0.27–1.04)	0.526
Overall	3288/3092	1.19 (0.86–1.63)	0.301

Abbreviations: OR, odds ratio; CI, confidence intervals.

**Table 5:** Subgroup analyses for the associations of VEGF +405C/G with susceptibility to lung cancer.

Subgroups	No. ofcase/control	Allele model(G allele vs.C allele)		Dominantmodel(GG+GC vs. CC)		Recessivemodel(GG vs. CC+CG)		Homozygousmodel(GG vs. CC)		Heterozygousmodel(GG vs. CG)	
		OR(95%CI)	*P*value	OR(95%CI)	*P*value	OR(95%CI)	*P*value	OR(95%CI)	*P*value	OR(95%CI)	*P*value
*Ethnicity*											
Caucasian	2332/1890	0.97(0.83–1.14)	0.732	1.01(0.69–1.49)	0.952	0.93(0.82–1.05)	0.245	0.96(0.66–1.39)	0.830	0.94(0.82–1.07)	0.363
Asian	722/990	1.04(0.64–1.69)	0.876	1.32(0.31–5.58)	0.709	0.66(0.39–1.12)	0.125	0.81(0.35–1.88)	0.629	0.55(0.22–1.34)	0.189
*Smoking status*											
Smoker	500/184	0.77(0.24–2.48)	0.657	0.47(0.09–2.50)	0.377	0.99(0.17–5.74)	0.988	0.55(0.04–7.36	0.652	1.19(0.19–7.30)	0.853
Non-smoker	346/255	0.86(0.64–1.15)	0.318	0.73(0.35–1.55)	0.413	0.85(0.57–1.25)	0.402	0.72(0.34–1.55)	0.406	0.87(0.58–1.31)	0.514
*Histological type*											
Adenocarcinoma	540/871	0.78(0.45–1.36)	0.383	0.51(0.18–1.48)	0.241	0.87(0.46–1.64)	0.673	0.53(0.15–1.88)	0.324	1.00(0.59–1.70)	0.999
SCC	736/871	0.77(0.40–1.49)	0.444	0.53(0.16–1.78)	0.306	0.84(0.41–1.73)	0.635	0.53(0.13–2.21)	0.386	0.95(0.53–1.71)	0.866
SCLC	274/871	0.66(0.43–1.00)	0.052	0.59(0.24–1.46)	0.251	0.49(0.25–0.94)	0.333	0.38(0.16–1.94)	0.335	0.53(0.25–1.12)	0.097
Overall	3035/2880	1.03(0.80–1.31)	0.840	1.23(0.60–2.50)	0.573	0.81(0.62–1.05)	0.108	0.92(0.62–1.37)	0.689	0.70(0.46–1.07)	0.099

Abbreviations: OR, odds ratio; CI, confidence intervals.

### *VEGF* −2578C>A Polymorphism and Risk of Lung Cancer

**Figure 2:**
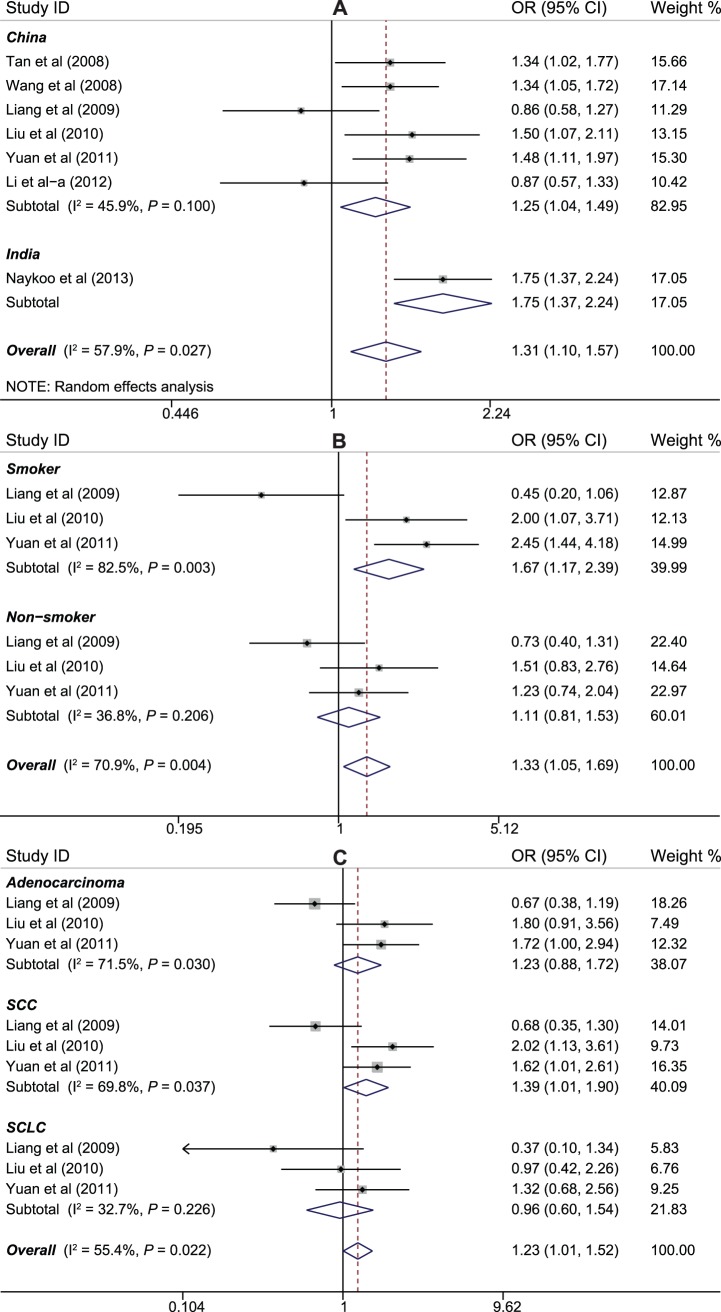
Forest plot of ORs for the association between *VEGF* −2578C>A polymorphism and susceptibility to lung cancer in subgroup analysis based on country of origin (A), smoking status (B), and histological type (C) under the dominant model.

A total of 7 studies with 22 data sets involving 1,596 cases and 1,857 controls were included in the pooled analysis. All subjects were of Asian ethnicity. Meta-analysis results showed that a statistically significant correlation was found between −2578C>A polymorphism and susceptibility to lung cancer in Asians under allele and homozygous models (for OR = 1.31, 95%CI = 1.10–1.57, *P* = 0.003; OR = 1.79, 95%CI = 1.30–2.46, *P*<0.001). We also performed a stratified analysis based on the geographic region of the studies; the results were persistent in both Chinese (for allele model: OR = 1.25, 95%CI: 1.05–1.49, *P* = 0.014; recessive model: OR = 1.88, 95%CI: 1.27–2.79, *P* = 0.002; homozygous model: OR = 2.00, 95%CI: 1.42–2.81, *P*<0.001; heterozygous model: OR = 2.06, 95%CI: 1.07–3.97, *P* = 0.032) and India populations (for allele model: OR = 1.75, 95%CI: 1.37–2.24, *P*<0.001; dominant model: OR = 1.26, 95%CI: 4.54–11.6, *P*<0.001; heterozygous model: OR = 1.08, 95%CI: 1.03–1.19, *P*<0.001) (Figure 2A). Additionally, a stratified analysis according to smoking status was performed using the information on packs of cigarettes smoked multiply by years of smoking; the variant allele is significantly correlated with increased risk of lung cancer among smoker subgroup(for allele model: OR = 2.18, 95%CI: 1.55–3.09, *P*<0.001; dominant model: OR = 1.67, 95%CI: 1.17–2.39, *P* = 0.005; homozygous model: OR = 7.31, 95%CI: 2.14–25.0, *P* = 0.002; heterozygous model: OR = 3.71, 95%CI: 1.06–13.0, *P* = 0.041), while not in nonsmokers (*P*>0.05 for all comparisons) (Figure 2B). Furthermore, we also stratified the case group by histological types and the data indicated the presence of the variant allele was the most strongly associated with SCC (for allele model: OR = 1.90, 95%CI: 1.43–2.52, *P*<0.001; dominant model: OR = 1.39, 95%CI: 1.01–1.90, *P* = 0.041; recessive model: OR = 4.87, 95%CI: 2.47–9.61, *P*<0.001; homozygous model: OR = 5.64, 95%CI: 2.79–11.4, *P*<0.001; heterozygous model: OR = 3.97, 95%CI: 1.94–8.10, *P<*0.001) (Figure 2C).

### *VEGF* −460C >T Polymorphism and Risk of Lung Cancer

**Figure 3:**
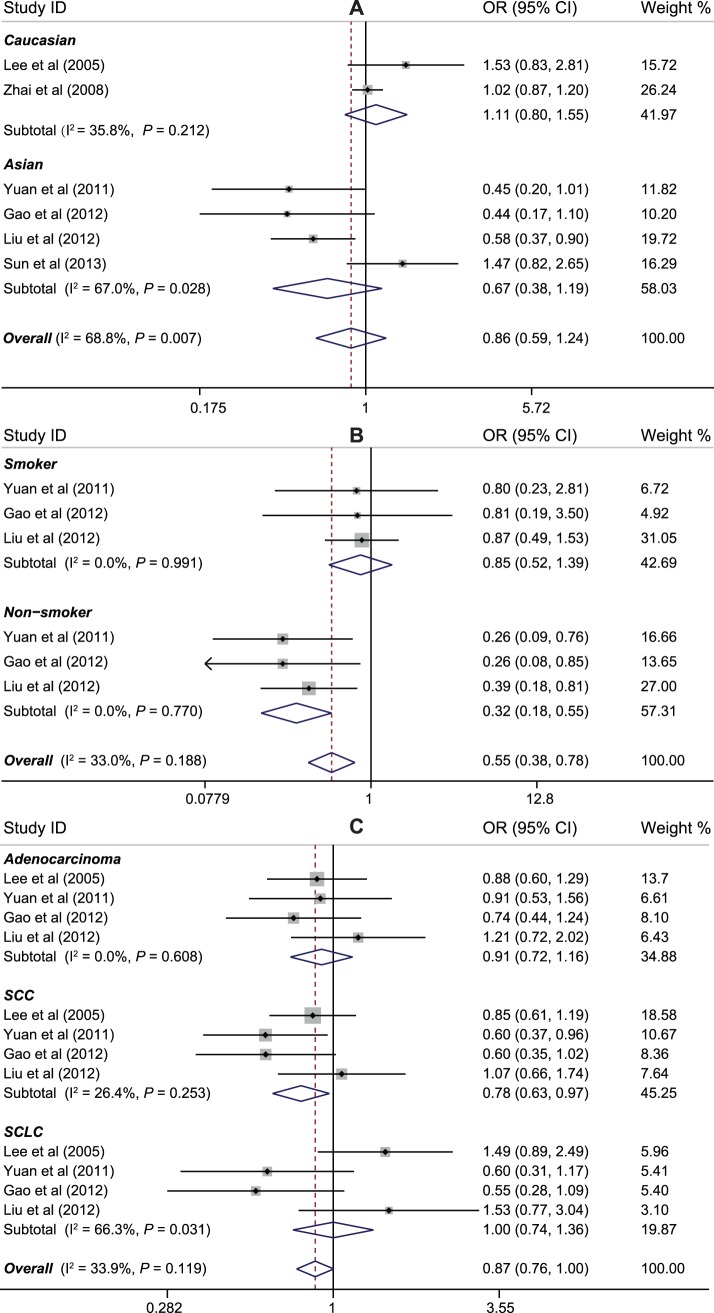
Forest plot of ORs for the association between *VEGF* −460C>T polymorphism and susceptibility to lung cancer in subgroup analysis based on ethnicity (A), smoking status (B), and histological type (C) under the dominant model.

*VEGF* −460C>T polymorphism was investigated in six studies with a total of 3,167 cases and 2,769 controls. There was no evidence of association between −460C>T polymorphism and lung cancer risk (for allele model: OR = 0.94, 95%CI: 0.79–1.12, *P* = 0.495; dominant model: OR = 0.86, 95%CI: 0.59–1.24, *P* = 0.415; recessive model: OR = 0.98, 95%CI: 0.73–1.32, *P* = 0.788; homozygous model: OR = 0.89, 95%CI: 0.60–1.33, *P* = 0.579; heterozygous model: OR = 1.01, 95%CI: 0.79–1.28, *P* = 0.969). Again, lung cancer cases and controls did not significantly differ in the subgroup analyses based on ethnicity (data not shown) (Figure 3A). However, when stratified analysis by smoking status was performed, a lower prevalence of the variant allele was observed among nonsmokers (for dominant model: OR = 0.32, 95%CI: 0.18–0.55, *P*<0.001; recessive model: OR = 0.35, 95%CI: 0.20–0.61, *P*<0.001) (Figure 3B). Additionally, further analysis on histological type was performed, and we found that −460C>T polymorphism was significantly associated with decreased risk of SCC (for allele model: OR = 0.81, 95%CI: 0.68–0.96, *P* = 0.013; dominant model: OR = 0.71, 95%CI: 0.48–0.97, *P* = 0.026; homozygous model: OR = 0.69, 95%CI: 0.46–0.94, *P* = 0.015; heterozygous model: OR = 0.81, 95%CI: 0.65–0.99, *P = *0.020), but not in adenocarcinoma and SCLC patients (*P*>0.05 for all comparisons) (Figure 3C).

### *VEGF* +936C>T Polymorphism and Risk of Lung Cancer

**Figure 4:**
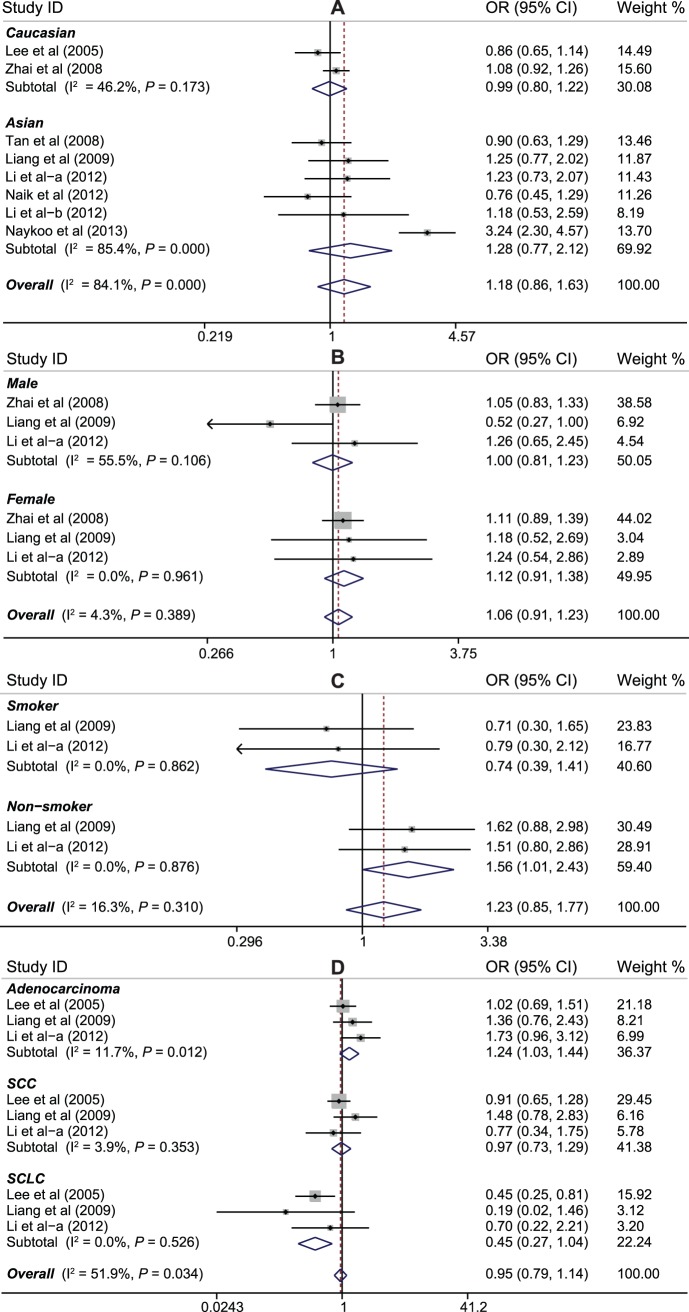
Forest plot of ORs for the association between *VEGF* +936C>T polymorphism and susceptibility to lung cancer in subgroup analysis based on ethnicity (A), gender (B), smoking status (C), and histological type (D) under the dominant model.

Eight studies investigated the association between +936C>T polymorphism and lung cancer susceptibility with a total of 3,288 cases and 3,092 controls. We did not find any association between +936C>T genotype and lung cancer risk under the dominant model (OR = 1.19, 95%CI: 0.86–1.63, *P* = 0.301), even when the groups were stratified by ethnicity (Caucasian: OR = 0.99, 95%CI: 0.80–1.22, *P* = 0.9372; Asian: OR = 1.28, 95%CI: 0.77–2.12, *P* = 0.338), gender (male: OR = 1.00, 95%CI: 0.81–1.23, *P* = 0.972; female: OR = 1.12, 95%CI: 0.92–1.38, *P* = 0.266), or smoking status (smokers: OR = 0.74, 95%CI: 0.39–1.41, *P* = 0.360; nonsmokers: OR = 1.56, 95%CI: 1.01–2.43, *P* = 0.052). Nevertheless, in the subgroup analysis on histological subtype, increased lung cancer susceptibility was shown among the adenocarcinoma subgroup (OR = 1.24, 95%CI: 1.03–1.44, *P* = 0.012) (Figure 4).

### *VEGF* +405C>G Polymorphism and Risk of Lung Cancer

A total of six studies involving 3,035 cases and 2,880 controls were included in the pooled analysis. The overall data did not show a marked association between +405C>G polymorphism and lung cancer risk in any genetic model, indicating that individuals with G allele might not have an increased lung cancer risk compared with those who carry wild-type C allele (for allele model: OR = 1.03, 95%CI: 0.80–1.31, *P* = 0.840; dominant model: OR = 1.23, 95%CI: 0.60–2.50, *P* = 0.573; recessive model: OR = 0.815, 95%CI: 0.62–1.05, *P* = 0.108; homozygous model: OR = 0.92, 95%CI: 0.62–1.37, *P* = 0.689; heterozygous model: OR = 0.70, 95%CI: 0.46–1.07, *P* = 0.099). Likewise, stratified analyses were also conducted according to ethnicity, smoking status and histological type of cancer. Unfortunately, there was no statistical difference in genotype distributions between cases and controls, and overall and different subgroups (*P*>0.05 for all comparisons; data not shown).

### Sensitivity Analysis and Publication Bias

Sensitivity analysis was performed to assess the influence of each study on the pooled ORs by omitting individual studies. The analysis results suggested that no individual study significantly altered the pooled ORs in *VEGF* −2578C>A, −460C>T, +936C>T, and +405C>G polymorphisms under the allele model (data not shown), which indicates that our studies were statistically accurate.

**Figure 5:**
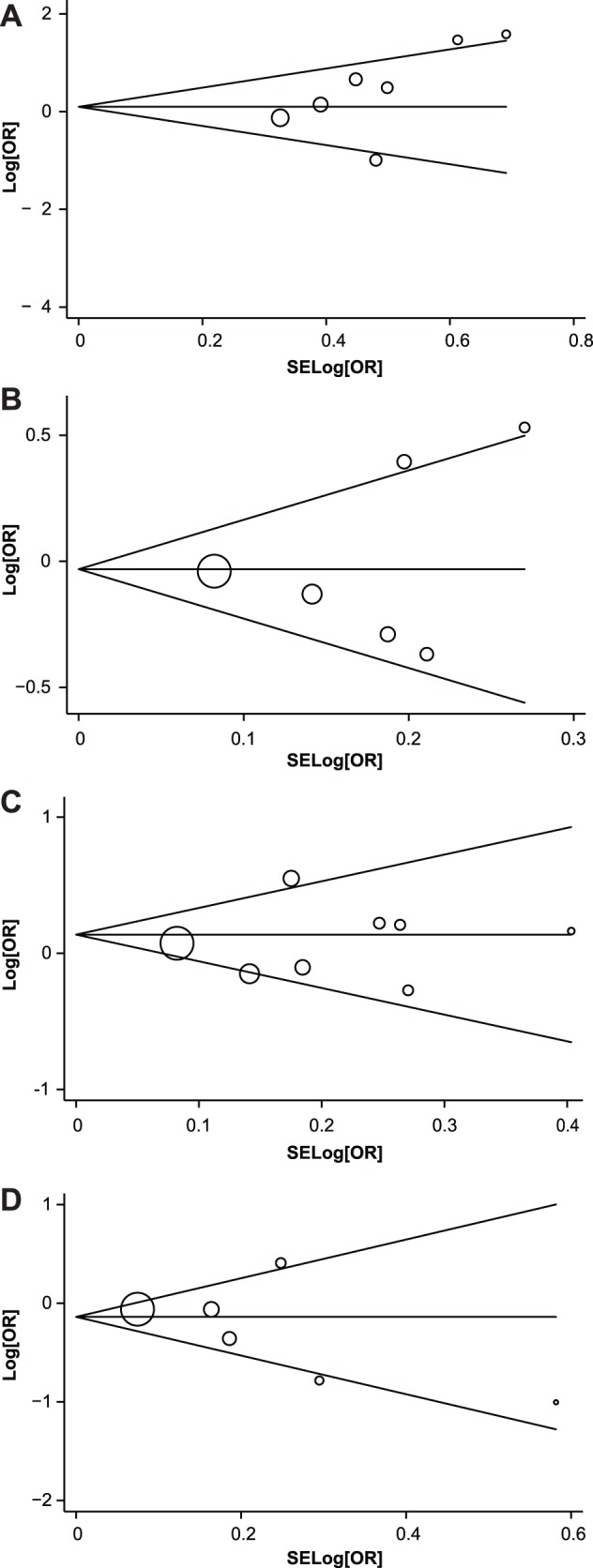
Funnel plots showed symmetric distribution. Log OR is plotted against the standard error of log OR for studies on *VEGF* −2578C>A (A), −460C>T (B), +936C>T (C) and +405C>G (D) polymorphisms. The dots represent specific studies for the indicated association.

Begger's funnel plot and Egger's linear regression test were performed on the metadata to assess publication bias of the individual studies. The shapes of the funnel plots did not reveal any evidence of obvious asymmetry in *VEGF* −2578C>A (A), −460C>T (B), +936C>T (C), and +405C>G (D) polymorphisms (Figure 5). Egger's test also displayed no significant statistical evidence of publication bias (−2578C>A: t = 0.99, *P* = 0.369; −460C>T: t = 0.53, *P = *0.623; +936C>T: t = 0.28, *P = *0.786; +405C>G: t = −1.08, *P = *0.339).

## Discussion

Evidence from preclinical and clinical studies shows that *VEGF*, as a predominant angiogenic factor in human cancers, plays a vital role in the carcinogenesis pathway, which has been proved to be a key step in tumor occurrence, progression and prognosis [12], [45]. Several functional polymorphisms of *VEGF* gene have been confirmed to be correlated with high levels of VEGF protein in cancer cells and high tumor angiogenic activity, and they also contribute to the susceptibility and severity of cancer, including lung cancer [36]. Although cigarette smoking is the major cause of lung cancer, only a small fraction of smokers develop this disease during their lifetime, which suggests that both genetic factors and lifestyle risk factors are modulating individual susceptibility to lung cancer risk. A study by Koukourakis et al. reported that non-small cell lung cancer patients with specific *VEGF* gene polymorphisms develop tumors with low *VEGF* expression and poor vascularization [46]. In recent years, the associations between *VEGF* and risk of lung cancer have been extensively investigated, obtaining conflicting results. Therefore, we employed a meta-analysis to explore a more precise evaluation for the associations. To our knowledge, this is the first meta-analysis on this topic.

The present meta-analysis, including 4,664 cases and 4,571 controls from 14 published case-control studies, explored the association between *VEGF* −2578C>A, −460C>T, +936C>T, and +405C>G polymorphisms and lung cancer risk. According to our pooled analysis, −2578C>A polymorphism may have a correlation with increased lung cancer risk. This finding may be biologically plausible since Koukourakis et al. observed that −2578CC was associated with lower VEGF expression and lower vascular density in lung cancer tissues compared to the −2578C C/A [46]. When lung cancer cases were stratified by histological subtype, the data indicated that the presence of −2578A was strongly associated with SCC, while similar finding was not observed in SCLC and adenocarcinoma. Although a research reported by Jin et al. demonstrated that −2578AA genotype was significantly associated with low histologic grade tumors [47], the reason for such a divergence of VEGF expression and angiogenic status in tumors of similar histologic type and differentiation remains obscure. Thus, more studies should be conducted to further examine the underlying mechanism. Furthermore, the stratified analysis according to smoking status revealed that −2578A is significantly correlated with increased risk of lung cancer among smokers, suggesting that this polymorphism may not be an independent risk factor, but perhaps an effect modifier that acts synergistically with smoking in lung cancer risk.

As for *VEGF* −460C>T polymorphism, the overall data did not show a marked association of this polymorphism with lung cancer risk in any genetic model, even in the subgroup analyses according to ethnicity. However, when stratified analysis by smoking status and histological type were performed, a lower prevalence of −460T allele was observed among nonsmokers, lung adenocarcinoma cases, and SCC cases. Some clinical evidence suggests that cigarette smoking may stimulate both angiogenesis and VEGF expression, which exacerbates the rapid cancer progression effect of angiogenesis [48], [49]. Thus, it is possible that cigarette smoke and *VEGF* activate multiple effects in lung cancer. For *VEGF* +936C>T, +405C>G polymorphisms, we found no overall association between these two polymorphisms or its interaction with smoking on lung cancer risk in any genetic model. When stratified analyses were conducted according to ethnicity and histological types of cancer, increased lung cancer susceptibility was only observed among the adenocarcinoma subgroup for +936C>T polymorphism, while there was no statistical difference in genotype distributions between cases and controls for any different subgroups for +405C>G polymorphism. Actually, there exist conflicting reports in some literatures regarding the exact function of the +405G/C polymorphism. Some clinical studies suggested that +405C allele has been associated with lower VEGF production, while some groups reported higher VEGF levels or even no association with +405C/C genotype [17], [50], [51]. Thus, whether these polymorphisms are truly functional requires further investigation through confirmatory studies and in vitro functional assays.

The current meta-analysis has several limitations that should be noted. First, the sample size in the present study was relatively small, so small, but potential, genetic effects may not be detectable. A small sample size may not have enough statistical power to explore the real association, especially in subgroup analysis. Additionally, as with other complex traits, lung cancer risk may also be modulated by several other genetic markers beyond *VEGF*, and our meta-analysis emphasized that elucidating the pathogenesis of lung cancer would demand an investigation into the association for many gene variants that may constitute distinct pathophysiological pathways. Third, we identified two studies from Caucasian populations and obtained no data from African populations, thus the two racial groups need to be further studied in the future. Therefore, the results should ideally be confirmed in further studies to strengthen the conclusions. Aside from the limitations listed above, our meta-analysis still has some strength. To the best of our knowledge, this is the first meta-analysis on the relationship between *VEGF* gene polymorphisms and lung cancer. We also explored inter-study variations by prespecified subgrouping of studies according to ethnicity, smoking status, gender, and histological type among cases. Furthermore, although this meta-analysis does not accommodate all previously published data, they are limited compared to the evidence that we generated.

In conclusion, this meta-analysis provides strong evidence that *VEGF* −2578C>A polymorphism is capable of increasing lung cancer susceptibility, especially among smokers and lung SCC patients. Additionally, for +936C>T polymorphism, increased lung cancer susceptibility was only observed among lung adenocarcinoma patients. In contrast, *VEGF* −460C>T polymorphism may be a protective factor among nonsmokers, lung adenocarcinoma and SCC patients. However, we did not find any association between +405C>G polymorphism and lung cancer risk, even when the groups were stratified by ethnicity, smoking status or histological type. More detailed and well-designed studies with larger population and different ethnicities are needed to further evaluate these associations.

## Supporting Information

Supplement S1 **PRISMA Checklist.**
(DOC)

Supplement S2 **Modified STROBE quality score systems.**
(DOC)
